# Effect of edge pruning on structural controllability and observability of complex networks

**DOI:** 10.1038/srep18145

**Published:** 2015-12-17

**Authors:** Simachew Abebe Mengiste, Ad Aertsen, Arvind Kumar

**Affiliations:** 1 Bernstein Center Freiburg and Faculty of Biology, University of Freiburg, 79104 Freiburg, Germany; 2 Computational Biology, School of Computer Science and Communication, KTH Royal Institute of Technology, Stockholm, 10044, Sweden

## Abstract

Controllability and observability of complex systems are vital concepts in many fields of science. The network structure of the system plays a crucial role in determining its controllability and observability. Because most naturally occurring complex systems show dynamic changes in their network connectivity, it is important to understand how perturbations in the connectivity affect the controllability of the system. To this end, we studied the control structure of different types of artificial, social and biological neuronal networks (BNN) as their connections were progressively pruned using four different pruning strategies. We show that the BNNs are more similar to scale-free networks than to small-world networks, when comparing the robustness of their control structure to structural perturbations. We introduce a new graph descriptor, ‘the cardinality curve’, to quantify the robustness of the control structure of a network to progressive edge pruning. Knowing the susceptibility of control structures to different pruning methods could help design strategies to destroy the control structures of dangerous networks such as epidemic networks. On the other hand, it could help make useful networks more resistant to edge attacks.

Graphs are a powerful conceptual framework to understand the behaviour of complex systems that involve a large number of interactions among their constituents^1^,^2^,^3^. In most chemical, biological, social and to some extent in engineering systems (e.g. electrical power grid), the intrinsic dynamics of the nodes is largely fixed, but the network structure (node count and edges between nodes) continuously changes due to edge failures, evolutionary modifications and activity-dependent short- and long-term plasticity mechanisms. Thus, it is reasonable to assume that part of the complexity of a system arises due to the structural perturbations in the underlying network. Interestingly, many graph descriptors may remain unaffected by structural perturbations, such as changes in the counts of nodes, edges, or the degree of nodes. That is, the invariance of some graph descriptors (e.g. shortest path length, global efficiency^2^) to structural perturbations could be a reason underlying the robustness and error tolerance of such complex systems^4^.

Therefore, there is a growing interest in relating graph properties to the system dynamics. Indeed, several graph theoretic descriptors of a complex system are correlated with some important dynamical properties of the system^3^,^5^,^6^,^7^,^8^,^9^,^10^. Despite this correlation between structure and dynamics, individual graph descriptors are in many cases insufficient to relate the structure of the graph to the system dynamics. This was most clearly observed in a recent attempt to relate network structure to the dynamics and stimulus response of a network of biological neurons^10^,^11^. One possible reason for this could be that a graph provides a static description of the system, in which the dynamics of the nodes is often ignored or simplified.

For practical applications, it is important to be able to steer a dynamical system to a desired state by forcing it externally (i.e. *controllability*) and to reconstruct the past dynamical trajectory from the current state of the system (i.e. *observability*). Thus, in a way controllability and observability define our understanding of a dynamical system.

Kalman^12^ showed that a full rank of the controllability and observability matrices ensures the controllability and observability of a system. Lin^13^ extended the notion of controllability to *structural controllability* and derived conditions that ensure the controllability of a network based only on the knowledge of connections without explicit information regarding their weights. Importantly, a minimum set of nodes, called *controls*, the stimulation of which would ensure full controllability of the system, can be obtained from the graph structure^14^,^15^,^16^,^17^. Similarly, the smallest possible set of nodes required to observe the entire system, called *sensors*, can also be found from the graph. Thus, controls (or sensors) may provide a better description of the graph, as they are more closely related to key aspect of the system dynamics, than other graph properties.

Here, we studied the effect of structural perturbations in complex networks on their control structure. While the robustness of the structural properties of complex networks is well established, it is not clear how their controllability changes upon edge deletion. Therefore, we investigated the robustness of control count to deletion of edges with or without fragmenting the network. First, we proved the equality of the numbers of controls and sensors for a given network. Thus our analysis of *controls* extend to *sensors* as well. Next, we developed new edge deletion strategies and analyse their performance in different types of networks. Specifically, we compared the effectiveness of these pruning strategies in affecting the numbers of controls or sensors in various types of random networks (such as Erdos-Renyi, small world, scale-free), social networks and biological neural networks – BNNs (large-scale connectivity of the macaque and mouse brains and the neuronal network in *C. elegans*). By comparing the changes in control count for different types of complex networks and BNNs, we show that in terms of their controllability, BNNs resemble scale-free better than small-world models. Finally, we introduced the notion of the cardinality curve of a complex network, not only to infer the performance of a pruning strategy, but also to extract important structural properties of the network and to compare different network structures.

## Methods

### Kalman controllability

Consider an n-dimensional linear system:


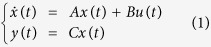


where *x* (dimensions *n* × 1) is the state vector of the system, *A* (dimensions *n* × *n*) is the adjacency or transfer matrix of the system, *B* (dimensions *n* × *m*) is the input projection matrix, *u* (dimensions 1 × *m*) is the input vector, *y* (dimensions *k* × 1) is the output vector of the system, *C* (dimensions *k* × *n*) is the readout interaction matrix of the system and *D* (dimensions *k* × *m*) is the contribution of input *u* to the readout. *m* ≤ *n* is the number of inputs and *k* ≤ *n* is the number of readouts of the system.

The Kalman controllability criterion states that a linear system is controllable if the controllability matrix, 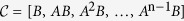 (dimensions *n* × *nm*), has full rank^12^. Similarly, the system is observable if the rank of the observability matrix, 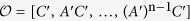 (dimensions *n* × *nk*), is n. The prime mark (′) denotes the transpose of the referred matrix. From these full rank conditions, we can ensure the observability and controllability of the system by appropriately choosing *B* and *C*.

### Structural controllability

The two matrices *A* = (*a*_*i*,*j*_) and 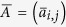 are called structurally equivalent if they have the same pattern of zero entries and non-zero entries. That is,


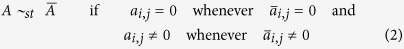


**Figure 1:**
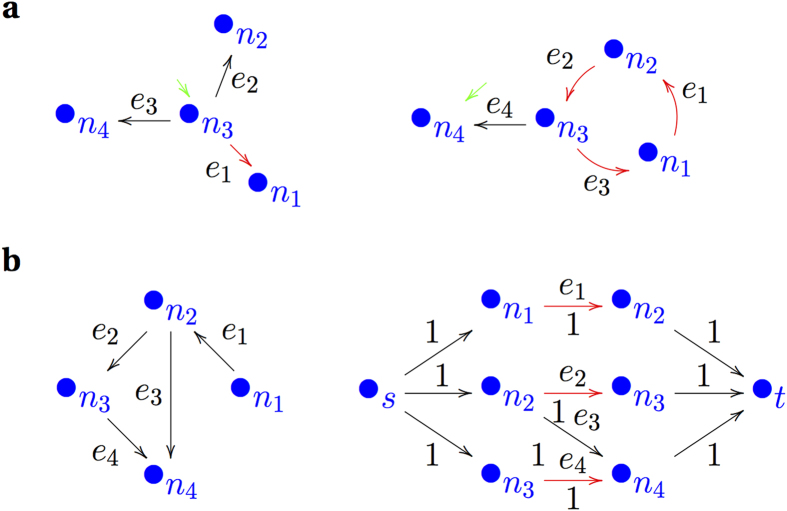
Schematic description of dilation, inaccessibility and maximum matching. Red arrows indicate the edges that constitute a maximum matching set. Green arrows indicate the external control input. (**a**) Dilation and inaccessibility problems are independent of one another. (**a** left) Example of dilation: an input at *n*_3_ could reach every node, but branching causes dilation. (**a** right) Example of inaccessibility: an input from *n*_4_ could not reach any other node however, there is no dilation. (**b**) Maximum flow, shown at (**b** right), is adapted to extract maximum matching set of a simple example (**b** left). The network is represented as a bipartite graph so that maximum flow from a global source node *s* to global target node *t* identifies a maximum matching set. Note that nodes *s* and *t* are not part of the original network.

A linear system is *structurally controllable* if there exists a controllable linear system with structurally equivalent connectivity and input matrices. *Inaccessibility* and *dilation* are two key properties of the network structure that determine the structural controllability of the system^13^ (cf. Fig. 1a). Inaccessibility occurs when a node cannot be reached from any input node. Dilation occurs when a parent node is driven to influence two or more children nodes at a time. To formally recall its concept on a linear system (interaction and input matrices (*A*, *B*)), we can consider a subset *S* of the system nodes and use the term *vertex in-cover* of *S*, denoted by *T*(*S*), to refer to the set of all nodes in *A* (from fellow nodes) or in *B* (from external input nodes) that are in-neighbours to at least a node in *S*. A dilation is said to occur in a system if there exists a larger subset *S* of system nodes than its vertex in-cover (i.e. *n*(*S*) > *n*(*T*(*S*))). A network without any dilation and inaccessibility is structurally controllable. One must therefore preclude dilation and inaccessibility by providing appropriate input to a set of controls in order to establish a control structure in a network.

We use the term *controls* to describe the smallest set of inputs, {*u*_*i*_(*t*)} in *u*(*t*) = (*u*_1_(*t*), …, *u*_*m*_(*t*))′ (eq. 1), that ensure the controllability of a linear system. The number of controls is denoted by *nCN*, which is also the number of control nodes or nodes in the system that are directly connected to the controls. The term *control structure* refers to a system with controls. Similarly, the *sensors* refer to the smallest set of outputs, {*y*_*i*_(*t*)} in *y*(*t*) = (*y*_1_(*t*), …, *y*_*k*_(*t*))′ (eq. 1), that ensure the observability of a linear system. The number of *sensors* is also the number of sensor nodes or nodes in the system that are directly monitored by the sensors. The term *observable structure* refers to a system with a set of sensors.

### Maximum-matching extraction algorithm

Because there is no analytical expression available to calculate the number of control nodes, we will use the concept of maximum-matching (MM) proposed by Liu *et al.*^14^. A *matching set* in a digraph is a set of edges that do not share their source and terminal nodes. In other words, if two edges (*a*, *b*) and (*c*, *d*) belong to a matching set *M* of a digraph, *a* ≠ *c* and *b* ≠ *d*. A matching set is called *maximal* if it can allow no other edge from the network. It is called *maximum* if it is maximal and with the maximum possible cardinality^18^,^19^. The nodes which are not targets of any edge in a matching set constitute a set of driving nodes for the controllability of the system. A similar set of nodes that are associated to a maximum matching (MM) set recruit a set of *controls*^14^, a set of driving nodes with the minimum possible number.

Here, we used Ford-Fulkerson’s maximum flow algorithm^20^ to extract MM sets. First, all nodes with non-zero out-degree are collected in one pool, while all nodes with non-zero in-degree are collected in another pool, such that a directed bipartite graph could be formed from the first pool to the second. Therefore, a node with out- and in-neighbours belongs simultaneously to the two pools. Secondly, a global source node and a global target node are added outside the pools, such that the source node is connected to all nodes in the first pool and all nodes in the second pool are connected to the target node. Every edge is assigned a unit weight to compute maximum flow. The selected edges in a maximum flow correspond to an MM set (cf. Fig. 1b). As a result of the multiple choice of edges of many networks for the maximum flow, there are different options of MM sets and hence controls (cf. Supplementary Fig. S1).

### Models of complex networks

To investigate the performance of the pruning strategies, we used biological, social and artificial random networks with different topologies. The biological networks includes the long-distance regional connectivity of the macaque brain^21^, the mesoscale connectivity map of the mouse brain^22^ and the neuronal network connectivity in *C. elegans*^23^. We used Google+^24^, Facebook^25^, Airport^26^, Amazon^27^, Peer-to-Peer^28^,^29^, EU-email^29^ and Wikipedia-vote^30^ networks in the category of social and related networks for our demonstration. The artificial networks included random^31^, small-world^23^ and scale-free networks (see the Supplementary Information: **Network generation procedures**). To create scale-free networks, three models were used; namely, Barabási-Albert, duplication divergence and local attachment models^32^,^33^,^34^,^35^,^36^. Table 1 summarizes the basic statistical features of the networks.

### Edge pruning strategies

We used *conditioned* and *unconditioned* pruning methods. In conditioned pruning approach, a pruning strategy takes care of keeping the network intact. That is, a pruning strategy will not delete an edge if its deletion results in fragmentation of the network. The unconditioned pruning approach does not obey this additional condition and could cause multiple components at any stage of pruning. A network of size *n* will have at least *n* − 1 edges at the end of a conditioned exhaustive pruning strategy, where as it will have no edge after an unconditioned pruning strategy. Under each of these two categories, we considered the following four exhaustive pruning strategies.

#### Random pruning

This model selects an edge randomly and removes it from the network (in unconditioned case) unless it fragments the network (in conditioned case). The deletion was performed repeatedly until every edge was deleted. This is the most intuitive and simple pruning strategy and full network connectivity is not necessary to implement it as edges are selected at random for deletion.

#### Out-pruning

In this pruning model, we started by randomly selecting a node and systematically removed its outgoing projections. Once the outgoing edges of the chosen node were exhausted, another node was picked randomly to repeat the pruning procedure. Similar to random pruning, the trimming procedure was performed until no edge is remained in the network (unconditioned) or until a last spanning digraph (conditioned). In this procedure, there is no special significance to the pruning of outgoing edges, and similar results would be obtained if pruning were performed based on the incoming edges. Like random pruning, we do not need to know the full connectivity of the network because pruning is performed on a node by node basis.

#### Ordered maximum matching (ordered-MM) pruning

This pruning method requires the knowledge of the full network connectivity. Here, we first ordered the edges *E* of the network after exhaustive extraction of maximum matching sets. The first maximum matching (MM) set takes all edges into account, while the subsequent ones are maximum matching sets of the remaining edges, excluding the already extracted sets.


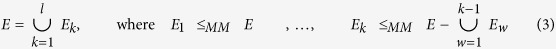


Hence, |*E*_*i*_| ≥ |*E*_*j*_| if *i* < *j*. Here, *A* ≤ _*MM*_ *B* denotes ‘*A* is a maximum matching set of *B*’. Clearly, *E*_1_, …, *E*_*l*_ form a partition of the set of all edges in the network. Each edge in the network belongs to some block, *E*_*k*_, in the partition. The relative rank, *rel. rank*, of an edge is then defined as the cardinality of the block it belongs to. For edge *e* ∈ *E*,


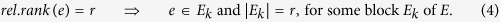


The deletion was performed in decreasing order of relative rank, i.e. all edges belonging to *E*_1_ were the first to be trimmed in any order. The procedure was repeated for all partitions according to the order of their indices.

We based this pruning strategy on the procedure we used to extract the controls. To understand, we need to recall the notion of *cactus* as proposed by Lin^13^. A cactus is a connected digraph consisting of non-overlapping *buds* (directed cycles) and a *stem* (simple directed path) such that a node in each bud has an incoming edge (called *distinguished edge*) from a distinct node on the stem. That is, two distinguished edges cannot share the same node in a cactus. Because a stem is a budless cactus, an isolated node in a network can be included as a degenerate cactus by its incoming edge from a control node. To design a control structure with minimum number of inputs, one needs to find some *minimum spanning cacti (MSC)*^13^ - that is, the smallest possible cacti that together span the network. The number of the MSC determines the number of controls. Moreover, all the edges of the cacti excluding the distinguished edges form the corresponding MM set to the control nodes, which is equivalent to *E*_1_ (eq. 3). That is, *E*_*i*_ contains all the edges in all the distinct directed cycles and simple paths in the set of MSCs at the *i*^*th*^ stage of network pruning (after the edges in 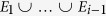 were removed). Thus, ordered-MM pruning could be regarded as an MSC destructor, because it targets edges of high relative rank, thereby forming an extreme case of pruning.

#### Resilient pruning

This pruning strategy is aimed at keeping the controllability profile of the network resilient to edge deletion. To this end, we designed three different methods. The first one was similar to the ordered-MM pruning but in the reverse order, i.e. in a partition {*E*_1_, *E*_2_, …, *E*_*l*_} of *E*, where |*E*_*i*_| ≥ |*E*_*j*_|whenever*i* < *j*, the order of deletion was performed from *E*_*l*_ to *E*_1_. That is, the edges that were part of the maximum matching set corresponding to the original network were pruned only after all other edges were systematically removed. Thus, by definition there was no change in the control structure of the network almost for the entire deletion. The supplementary information includes a brief description of the three different ways of resilient pruning implementation (see the Supplementary Information: **Alternative implementations of resilient pruning**).

Both ordered-MM pruning and resilient pruning required ordering all edges based on their relative rank (cf. eq. 4). According to the partitioning of edges as described in eq. 3, the cardinality of a maximum matching set determines the relative rank of the included edges, i.e. the higher the cardinality, the higher the rank order of the contained edges. While ordered-MM pruning was performed in decreasing order of relative rank, resilient pruning was performed in increasing order. Random pruning and out-pruning, however, required no knowledge of the relative rank of network edges. By design, resilient pruning is an extreme case in which a network would be sparsened without any change in the control node set, except when deleting the final few edges. By contrast, ordered-MM pruning is another extreme strategy that should result in a maximal change in the driver node count.

Because the maximum matching set is not unique (cf. Supplementary Fig. S1) and depends on the initial conditions, we estimate the change in the controllability configuration for 50 different realizations of each pruning strategy and for each network type. We mainly performed exhaustive pruning. For each edge deletion, a set of controls was extracted *m* times corresponding to each deletion and their number, *nCN*, was stored. Therefore, a single realization extracts controls *m* times, corresponding to each deletion. To enhance random sampling, the network nodes were reindexed for each realization. In other words, each strategy first chose a random permutation matrix *P* to re-index the connectivity matrix *A* as *P*^−1^*AP*, such that nodes and edges could have different IDs for each realization.

### Quantitative measures of pruning

#### The rate of change of control count (*R*_*C*_)

To quantify how fast a pruning strategy changes the number of controls *nCN*, we calculate the slope (*R*_*C*_) with respect to the fraction of deleted edges. *R*_*C*_ counts the changes in elevation between the initial and final edge deletion stage of interest. The mean slope of the overall performance of a pruning strategy is simply the slope from the first to the last deletion.


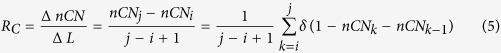


where, *∆L* stands for the change in the number of edges. *nCN_i_* denotes the number of controls after the deletion of *i* edges. *δ* refers to the Kronecker delta function.

#### Pruning Damage Index (*PDI*)

To compare the overall damage caused by any pair of pruning procedures, we define the *pruning damage index (PDI)* for a pruning strategy (*ps* ∈ Random, Out-, Ord–MM, Resilient), from *d*_*i*_ to *d*_*f*_ deletion of edges as the normalized area between the pruning curve and the baseline (i.e. the horizontal line of the initial control size *nCN*_0_) of the network. With Δ*d*: = *d*_*f*_ − *d*_*i*_ + 1 and *N*: = size of the network,


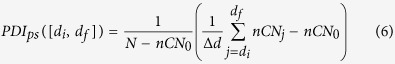


when *d*_*i*_ = 0 and *d*_*f*_ is the number of edges in the network, *PDI* is referred to as the *exhaustive pruning performance index (ePDI)*, to quantify the overall damage of an exhaustive pruning strategy.

## Results

Here, we investigated the robustness of various complex networks and BNN to structural perturbation. Specifically, we estimated the control structure by the number of controls that ensured structural controllability of the system. While the emphasis is on the controllability, it is directly related to the notion of ‘structural observability’ which allows for the reconstruction of system trajectories from a small number of sensors. We first proved that the numbers of controls and sensors are equal, thereby, we argue that our results are also valid for the estimation of sensors in a complex network.

### Equality of optimal number of controls and sensors

Theorem: *The numbers of controls and sensors required for controllability and observability of a linear system are equal.*

The proof follows from the well-known theorem of controllability-observability duality. If we have a pair of linear systems as:


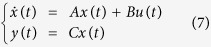



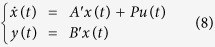


the duality theorem implies that the controllability grammian 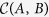 of the first system (eq. 7) and the observability grammian 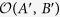 of the second system (eq. 8) are equal.


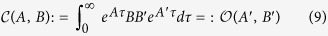


Structurally, it further reveals that the number of controls of a linear system with system matrix *A* is equal to the number of sensors of a linear system with *A*′ as system matrix. It is clear that the numbers of controls of the two systems are equal as their system matrices are transposes of each other. Therefore, the number of controls and the number of sensors are equal.

This result ensures that any statement made about the optimal number of controls is also valid for the optimal number of sensors. Moreover, the number of sensors or controls cannot be less than the number of *leaf nodes* or *source nodes* of the network. A leaf node or simply a leaf in a network is a node without any outgoing edge, while a node in a network is called a source node if it does not have any incoming edge.

### Performance of the pruning strategies on complex random networks

To understand how changes in the network structure affect the controllability configuration of complex artificial and biological networks, we estimated the change in the number of controls (*nCN*) as we progressively pruned the edges and, thereby, sparsened the network. Because, structural controllability requires full accessibility^13^, it is expected that the number of controls will increase as the edges are pruned upon sparsening. However, the maximum matching of an adjacency matrix depends also on the probability of pair-wise connections and directed cycles of multiple nodes. In fact, complex networks with the same average connection probability show different controllability configurations, depending on the structure of the network^14^,^32^. Therefore, we estimated the control profile of several different types of complex artificial networks for progressive pruning of the edges.

Because there is no standard pruning strategy available, we first designed four different pruning strategies (see Methods). Two intentional edge-attack strategies were derived from the procedure we used to extract the control nodes (ordered-MM and resilient pruning). These two pruning strategies require knowledge of the full connectivity, both to initiate the pruning process and to calculate the controllability profile. When the full network connectivity is not available, pruning could be implemented by random deletion of known edges (*random pruning*) or by progressively removing projections of randomly chosen nodes (*out-pruning*).

**Figure 2:**
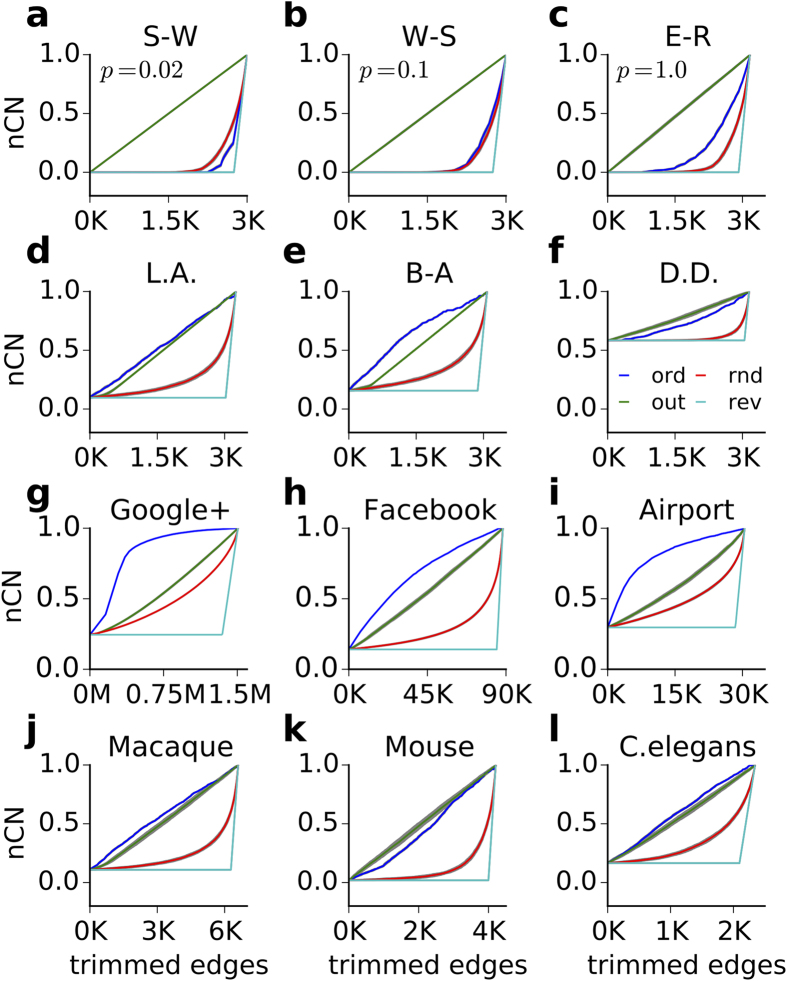
Effect of the four pruning strategies in changing the control count in different network types. (**a**) Effect of four different pruning strategies on a small-world network. The green, blue, red and cyan represent the effect of out-pruning (out), ordered-MM pruning (ord), random pruning (rnd), and resilient pruning(rev), respectively. Each of the curves shows the mean of 50 different realizations bounded by a gray region, representing the corresponding standard deviation. For most of them, the gray region is very small narrow to be visible. (**b–l**) Same as in panel (**a**) but for different types artificial, social and biological networks. The name of the network is mentioned on the respective panel. (**a–c**) Watts-Strogatz spectrum of directed networks with respective randomising probabilities of 0.02, 0.1 and 1, hence small-world (S-W), intermediate (W-S) and Erdos-Renyi (E-R) random networks. The panels (**a–f**), refer to artificial networks of size 250 and connection density of nearly 5%. (**g-i**) Social networks from Google+, Facebook and Airport connections. (**j–l**) BNNs: large-scale connectivity of the macaque brain, the meso-scale connectome of the mouse brain and the complete neuronal connectivity of *C. elegans*. The color descriptions of all curves is shown in panel (**f**) as green, blue, red and cyan - it represents the effect of out-pruning (out), ordered-MM pruning (ord), random pruning (rnd), and resilient pruning(rev), respectively.

We studied the effect of the four pruning strategies on complex networks with average connection probability of ≈5%. Complex networks with higher connection probability usually have very few controls^14^ or they show no change in the control structure for the first stage of edge deletion. Most complex artificial networks studied here showed considerable robustness to random pruning, even if nearly fifty percent of the edges were pruned (cf. Fig. 2a–l red traces). In random, small-world and other Watts-Strogatz complex networks with ≈5% or more average connection probability, there are many more edges which are not included in the maximum matching set, and the edges which are part of the maximum matching set could be replaced by other edges (see section **MM cardinality curve**). Therefore, even for up to 50% pruning (average connection probability ≈2.5%), the cardinality of a maximum matching set remained largely unaffected in these networks. However, as the networks became progressively sparser, the network reached a structure where the edges inside the MM set could no longer be replaced by other edges, resulting in a sharp increase in the number of controls (see also Supplementary Fig. S2).

While in random pruning, we randomly chose the edges to be pruned, in out-pruning, we randomly selected a node and progressively removed its outgoing edges. Out-pruning promoted the formation of leaves without much decrease in the average connection probability in the network. If the transpose was considered (‘in-pruning’), many sources tended to be formed. Leaves, by definition, cannot control other nodes and, therefore, increase the number of controls. Similarly, source nodes, without any incoming edge, are evidently part of any set of controls. Thus, the out-pruning strategy is very effective in increasing the control count in complex networks with different topology and connection density (cf. Fig. 2). In addition, the efficacy of out-pruning in increasing the control count could be further improved if, instead of selecting nodes at random, nodes were chosen based on their out-degree, in-degree or degree (cf. Supplementary Fig. S3). These results hold for both conditioned and unconditioned pruning (see the section **Conditioned pruning**).

Different classes of complex networks show different sensitivities to the edge pruning strategies other than the resilient pruning. Most networks with power-law or exponential degree distribution (such as local attachment, Barabási-Albert) show the highest sensitivity to ordered-MM pruning, as expected (cf. Fig. 2d,e). In scale-free networks, ordered-MM pruning is at least as effective as the out-pruning in altering the controllability configuration (cf. Fig. 2f). However, in the Watts-Strogatz (WS) spectrum of directed networks, ordered-MM pruning is not the most severe in changing the controllability configuration of the networks (cf. Fig. 2a–c). To understand this, it is important to remember that small-world networks are essentially ring lattices with a small proportion of rewired edges. Ring lattices have giant rings or long paths, which result in multiple disjoint maximum matching sets of same cardinality. Continuous extraction of MM sets, therefore, gives rise to many sets of nearly equal cardinality and, hence, the control count does not change with ordered-MM pruning. In Erdos-Renyii networks, ordered-MM pruning, although performing better than in small-world networks, remains less effective than out-pruning due to basically the same reason, that is, the cardinalities of most MM sets are equal or show only little difference (MM cardinality curve).

### Performance of the pruning strategies on social networks

**Figure 3:**
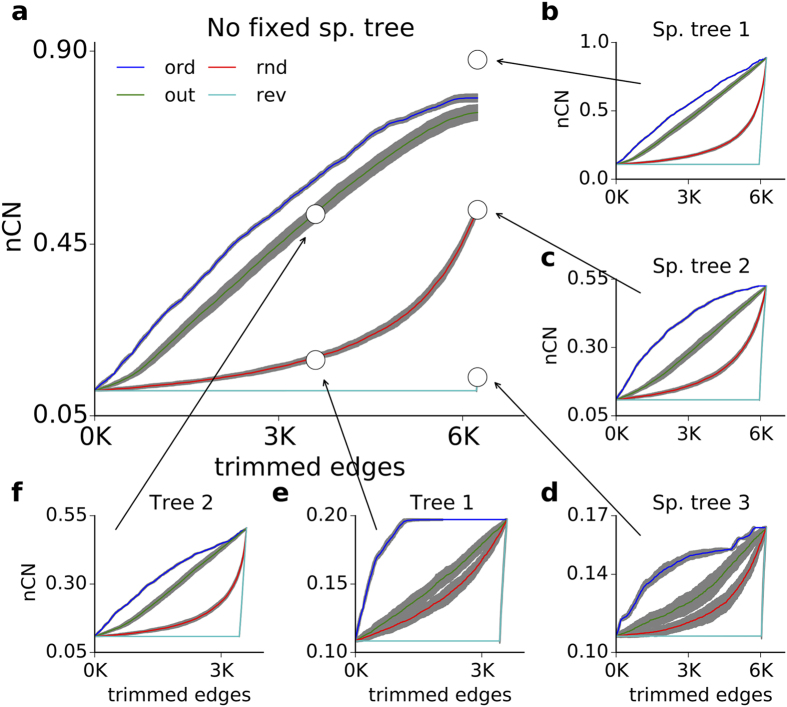
The effect of protecting a spanning digraph from deletion. (**a**) Effect of the conditioned pruning strategies on the control node count in macroscopic connectivity of macaque brain. The white circles show five different network structures (spanning digraph) which could either be achieved through a specific pruning process (when the white circle lies on the four traces) or arbitrarily (when the white circle lies on the four traces). **(b–f**) The effect of pruning process on the original macaque brain network without affecting the chosen spanning digraph marked by the white circles. Note that, by preserving a specific spanning digraph, each pruning strategy results in same (*R*_*C*_ eq. 5), unlike the in panel (**a**) where we did not care to preserve any specific spanning digraph.

Among real-world networks, social networks rapidly undergo large structural changes. Because most social networks are scale-free, we would expect ordered-MM pruning would be most effective in increasing the *nCN*. To test this, we investigated seven different types of social networks (see Methods). Indeed, the effect of different pruning strategies on social networks is similar to their effect on artificially constructed scale-free networks. The Gnutella peer-to-peer network (dated August 31 2002) is not scale-free and, therefore, out-pruning, instead of ordered-MM pruning, was the most effective in increasing the *nCN* count (cf. Table 1, Fig. 3).

### Performance of the pruning strategies on BNNs

Biological neuronal networks (BNNs) form another class of highly dynamic networks. Activity-dependent structural plasticity, reorganization of synapses during development and learning, and structural modification related to brain disease render the structure of BNNs highly dynamic^37^. Therefore, studying the controllability of BNNs could provide key insights into development-related pruning and improve our understanding how network function deteriorates upon loss of synapses in neurodegenerative diseases.

We found that in BNNs random pruning procedure results in an exponential dependence of the number of controls on the average degree of the network (cf. Supplementary Fig. S4). In BNNs, independent of the size of the network (or the species), both out-pruning and ordered-MM pruning result in similar changes in the controllability configuration (cf. Fig. 2g–i). This is similar to the scale-free networks and networks with exponential degree distribution (compare Fig. 2d–i). That is, BNNs are more similar to scale-free networks or to networks with exponential degree distribution than to small-world networks in terms of their sensitivities to edge-attacks (cf. Fig. 2d–i).

### Conditioned pruning

So far, the four pruning strategies were performed without any additional condition, so that the network could ultimately lose all its edges (cf. Fig. 2). We obtained similar results when we imposed a restriction on the exhaustive pruning strategies, i.e. not to fragment the networks (compare Supplementary Fig. S5 and Fig. 2).

The only difference between the ‘fragment’ and the ‘no fragment’ conditions was that in the former, the pruning process continued until the last edge in the network, whereas in the latter, pruning stopped when the network became a tree. That is, under the ‘no fragment’ condition, an exhaustive pruning strategy sparsened a complex network to one of its *spanning directed trees (SDT)* as its terminal structure. A spanning directed tree (SDT) of a network is a spanning subgraph without any cycle. Moreover, the SDTs resulting from different pruning strategies were different in terms of their control structure. For example, a terminal SDT of the macaque brain connectivity by the random pruning strategy required more controls than a terminal SDT by resilient pruning (compare the end points of the pruning lines in Fig. 3a or Supplementary Fig. S5).

To understand whether the terminal SDT affects the change in the control structure as a function of the pruning strategy, we further restricted the pruning criterion and imposed the additional condition that a predefined spanning subgraph should be preserved throughout the pruning process, such that all pruning strategies ended with that subgraph. Concepts of maximum-leaf spanning tree^38^ or a tree with minimum vertex cover^39^ can be used to create an SDT of relatively many controls. In contrast, a long SDT with few branches can be chosen for few controls. We used the large-scale inter-areal network of the macaque brain for demonstration and applied the four pruning strategies towards five different spanning subgraphs (Fig. 3a white circles. The SDT in Fig. 3b is a hub-based SDT (cf. Supplementary Information: **Hub-based spanning directed tree**.) The SDTs in Fig. 3c–d are the preferential SDT destinations of the exhaustive random and resilient pruning procedures, respectively. 

Preserving only edges as few as the network size during pruning had a tremendous effect on the overall performance of the pruning strategies. If the network was pruned towards an SDT with more branching Fig. 3b all pruning strategies had a stronger effect on the rate of change of the fraction of the control count (*R*_*C*_). If it was pruned towards an SDT with long paths Fig. 3d the *R*_*C*_ remained low. If an SDT with intermediate structure was preserved Fig. 3c *R*_*C*_ also became moderate.

We considered two more spanning digraphs with 3,000 edges, visited during random pruning Fig. 3e and out-pruning Fig. 3f respectively. In the former, ordered-MM pruning and random pruning were forced to slow down and speed up, respectively, to meet the pace of out-pruning. In the latter, both ordered-MM and out-pruning were forced towards the random pruning trajectory. If a spanning digraph was selected anywhere along the horizontal part of the resilient pruning curve in (Fig. 3a cyan trace), all edge attacking strategies under the restriction of this chosen spanning digraph would end up to be ineffective and overlapped in a horizontal line.

In general, pruning under the restriction of a final network structure often resulted in a consistent order of performance. For example, in the macaque network, ordered-MM pruning was the most effective in increasing the control count, followed by out-pruning, random pruning and resilient pruning, respectively. However, the rate of change of *nCN* was different for each pruning strategy and the type of the preserved spanning subgraph. Importantly, these results also showed that the efficacy of a certain pruning strategy depends on both the initial and the final network structure. Unlike previous suggestions, in these networks at any stage of pruning, the number of control nodes could not be predicted from the count of strongly connected component (SCC)^40^ or the nodes with degree less than three^15^ (cf. Supplementary Fig. S6).

**Figure 4:**
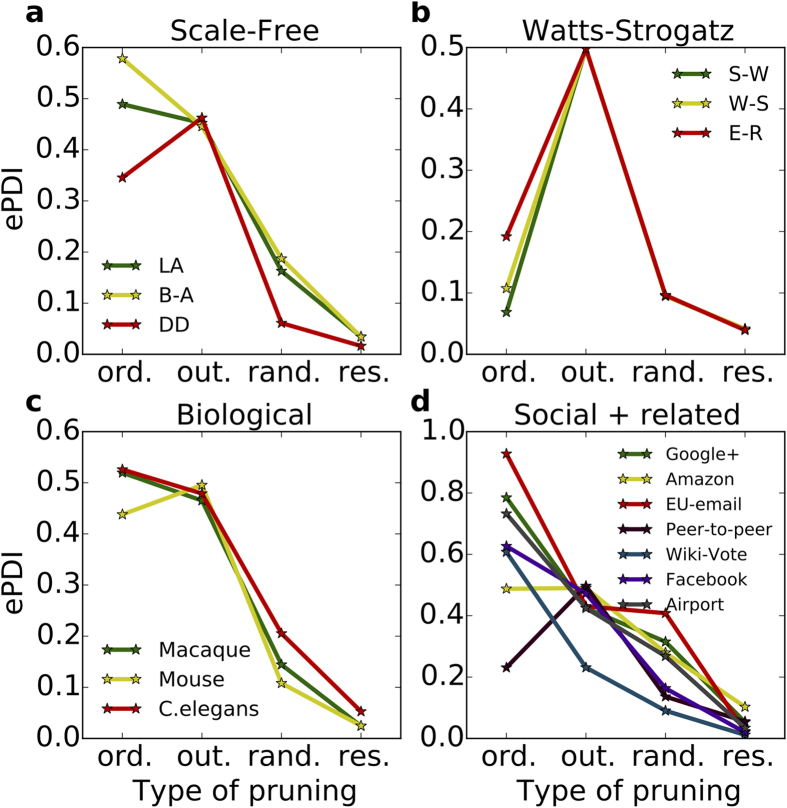
Exhaustive pruning damage index (ePDI) for different complex networks. The overall damage indices of the four pruning strategies ordered-MM, out-, random and resilient pruning strategies (ord., out., rand., res.) are shown for synthetic (three scale-free (**a**) and three Watts-Strogatz models (**b**)) and real networks (three biological neuronal networks (**c**) and seven social and related networks (**d**)).

To measures the sensitivity of a network control structure to the pruning strategies between any two stages of edge attacks, we evaluated the pruning damage indices of three scale-free, a small-world, a random, an intermediate W-S, three biological and six social and related networks (cf. Fig. 4). The biological, social and related networks studied here show similarity with the scale-free network models with respect to their sensitivity to the pruning methods. Gnutella peer-to-peer network (See Table 1 and Fig. 4) exceptionally is more similar to the Watts-Strogatz models. It is a good example of a social network that is not scale-free.

### MM cardinality curve

In all complex networks studied here, the order of efficacy of three of the four pruning strategies, with the exception of ordered-MM pruning, is preserved for the different networks (cf. Fig. 2, Supplementary Fig. S5). To better understand the efficacy of ordered-MM pruning in changing the control structure of a network and why it behaves differently from the other three pruning strategies, we designed the cardinality curve.

Exhaustive extraction of maximum matching (MM) sets provides a way to partition the set of edges *E* in a network (cf. Methods: **ordered-MM pruning**) i.e. 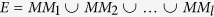  , where *MM*_1_: = a maximum matching  set  of  *E* and  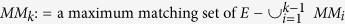. The indices 1, …, *l* of the blocks *MM*_1_, …, *MM*_*l*_ of the partition indicate the order of extraction. The cardinality of *MM*_*k*_ decreases or remains the same as *k* increases, i.e. |*MM*_1_| ≥ |*MM*_2_| ≥ … ≥ |*MM*_*l*_|. The cardinality curve refers to the cardinalities of MMs across their indices.

An edge in a network is *critical* in structural controllability if in its absence the network requires an additional control node^14^. All the critical edges, if any, belong to any MM set. One can in fact identify all the critical edges of a complex network from just an MM set by checking each edge for criticality using a simple algorithm: delete edge, check the control count and add the edge back. However, many networks exist without or few critical edges (cf. Table 1). Therefore, MM set could be viewed as a generalization of critical edges, a few edges that likely cause high increase in the number of controls if removed. Moreover, partitioning the network edges in terms of successive MM sets is a way of sorting edges relatively based on their criticality.

**Figure 5:**
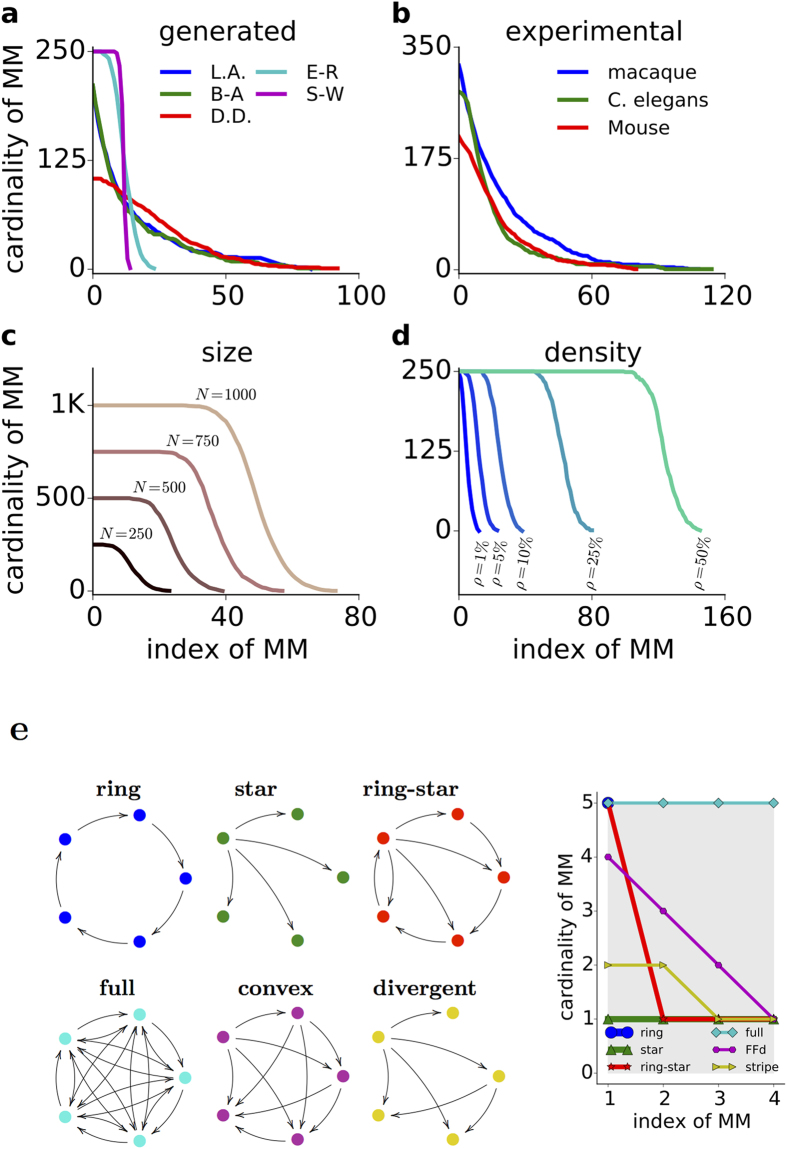
Cardinality curves. The cardinality curve plots the cardinalities of MM sets against their indices of extraction, from earliest to latest order. It enables to compare the performance of ordered-MM pruning with that of the other pruning strategies. The steeper the curve, the higher the performance of ordered-MM pruning. (**a**) Cardinality curve of the three scale-free. (**b**) Same as in (**a**) for the biological networks. (**c**) Cardinality curve of random networks with different size and fixed connection probability (*ρ* = 5%). Zero slope implies zero effect of ordered-MM pruning on *nCN*, as in SW (small-world) and random (E-R). (**d**) Cardinality curve of a random network (N = 250) for different connection probabilities (*ρ*). (**e,** left) Six basic network types. Convex refers to a network whose adjacency matrix is a lower triangular matrix. Divergent also refers to a lower triangular adjacency matrix, but with some columns set to zero (i.e. some nodes have zero out-degree). (**e,** right) Cardinality curves of six basic network types. The gray region covers the density of the full network without self connections – it helps to compare the connection density of the other networks from the area bounded under their cardinality curves. The cardinality curve reveals also important network features such as the degree heterogeneity by its steepness, the connection density by the area under it, the existence of hubs by its horizontal length, and the existence of long paths or cycles by its height.

Different artificial and biological networks show a decreasing sigmoid cardinality curve, with different threshold and slope (cf. Fig. 5a,b). The flat part of the cardinality curve shows that a network has multiple MM blocks of equal cardinality. Indeed, if there is a horizontal segment as part of the cardinality curve from *j* to *k* stages of MM extractions, the length *k* − *j* + 1 of the segment reveals the number of edge-disjoint sets of minimum spanning cacti (MSC) in the updated network with edge set 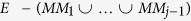. This implies that the network has different options of controls sets because it can use the disjoint sets of edges, thereby increasing the network robustness to ordered-MM pruning (e.g. Fig. 5a, purple and cyan traces). On the other extreme, a monotonically decreasing cardinality curve indicates a rapid change in the control count, thereby making ordered-MM pruning the most effective as, for instance, observed in scale-free networks (e.g. Fig. 5a, blue and green traces).

A larger size (Fig. 5c) and higher connection density (Fig. 5d) in E-R networks contribute to a longer flat beginning of the cardinality curve. The similar decaying part of the cardinality curve indicates that the effect of ordered-MM pruning of E-R networks is independent of network size and connection density.

The cardinality curves of the BNNs (Fig. 5c) are similar to those of scale-free networks (D.D. and B.A.). This emphasizes once more that BNNs are more similar to scale-free networks than to small-world networks, the cardinality curves of which start with a distinct flat part (cf. Fig. 5a,b).

### MM cardinality curve reveals structural network properties

In addition to providing an intuitive understanding of the efficacy of ordered-MM pruning, the cardinality curve also reveals different structural network properties. In fact, different network motifs result in characteristic cardinality curves (Fig. 5e).

On the one hand, the maximum value of the cardinality curve (*CC*_*max*_) gives an estimate of the *diameter* of the network graph (Fig. 5e). The diameter of a graph is the maximum finite distance between a pair of nodes in a network^41^. The number of MMs (*N*_*MM*_), on the other hand, gives an estimate of the maximum in-degree or out-degree of the network, whichever is larger. The *CC*_*max*_ and the number of MMs could be as large as the network size, *N*. When the *CC*_*max*_ = *N*, there is a giant directed cycle in the network. Moreover, the higher the value of *CC*_*max*_, the smaller the number of source and leaf nodes in the network.

Similarly, *N*_*MM*_ provides insights on the degree distribution of the network. If *N*_*MM*_ is small compared to the network size, all nodes have only few neighbours. If *N*_*MM*_ is large, there is at least one hub in the network. Moreover, any point on the cardinality curve indicates the number of nodes (y-axis) and their corresponding *max* (in-degree, out-degree) (x-axis). Finally, the area under the cardinality curve reflects the total number of edges and it can, hence, be used to estimate the connection density of the network.

The cardinality curve derived from the MM of a network is thus a very useful tool, not only to estimate the efficacy of different pruning strategies, but also to infer structural features of the network. It could be used to classify different complex networks.

## Discussion

To the extent the dynamics of a complex system can be described by linear dynamics, structural controllability is a powerful tool to relate the network structure to the activity dynamics. Here, we quantified the robustness of complex networks (artificial, social and biological networks) in terms of their controllability. We studied the evolution of the control structure as we progressively pruned the network according to each one of four different strategies (Fig. 2). We show that the vulnerability of complex networks to edge-attack depends on the pruning strategy. Each of the four pruning strategies was performed exhaustively so that we could see not only the effect of ordinary or critical edge removal on the control structures of the original networks, but also on the successively updated networks. Two of our pruning strategies exploited the backbone of an optimal control configuration, minimum spanning cacti. While ordered MM pruning continuously targeted the edges in minimum spanning cacti^13^, resilient pruning preserved them for later stages of deletion. We note that we here focused on minimizing the count of the controls. This meant that the identity of the controls could be different at each stage of pruning. Our results about the controllability of the network can be extended to their observability, because we have proved the equivalence of controls and sensors.

The control profile of most of the networks is robust to random edge deletion. However, it is possible to introduce a bigger change in the control profile by removing specific edges. For instance, intentional edge-attacks based on targeting the nodes with highest degree^42^ or higher betweenness centrality^43^ are more effective in changing the control profile than random edge-attacks. However, as we have shown here, it is possible to design other forms of intentional edge-attacks that either enhance or preserve the control node count. Resilient and ordered-MM pruning strategies are the two extreme forms of intentional edge-attacks, as they are derived from the very process that we used to identify the control nodes. While resilient pruning preserves the control node count, ordered-MM pruning induces a maximal increase in the control node count, except in the case of networks with high small-world index.

After an edge attack, a subset of nodes could still remain controllable given the original set of controls. Such nodes could be identified based on their reachability from the original control nodes^44^. By contrast, after every edge attack we re-calculated the set of controls to ensure the controllability of the full network. Another approach to restore the controllability of the network could be to add new edges systematically by interconnecting long paths and form longer paths^45^.

The change in the control node count could be transformed to calculate the ‘cardinality curve’. This new tool not only helps us quantify the efficacy of a pruning strategy to change the control structure of the network, but it could also be used to classify the network as scale-free, Erodos-Renyii, small-world or intermediate.

In fact, our comparison of the sensitivity of various network types to pruning suggests that BNNs are more similar to scale-free networks than to small-world networks. In the last decade, a number of studies have suggested that the BNNs in different species resemble the small-world topology^46^,^47^,^48^. The small-world topology is interesting because it improves the network communication while minimising the network wiring length^47^. However, recent analyses suggest that BNNs may not be as similar to the small-world networks as previously thought^49^,^50^. For instance, the small-world index of BNNs is much smaller than expected for small-world networks^49^,^51^. In general, classification of directed graphs based on their small-world index is problematic because the average path-length is not well-defined for many directed graphs (see Supplementary Information: **Network generation procedures**). The cardinality curve of various networks (Fig. 5) reveals that BNNs are, in fact, more similar to scale-free networks or to networks with exponential degree distribution than to small-world networks (cf. Fig. 2).

One of the best example of progressive pruning is a BNN which undergoes massive pruning during early development^52^,^53^,^54^ or in degenerative brain diseases such as Alzheimer’s disease (AD)^55^,^56^. Because different pruning strategies affect the *nCN* in a different manner, it is likely that the pruning strategy during development is different from the pruning that happens in AD. In general, studying the temporal evolution of the structural controllability of biological and other physical networks could provide insights into the structural organization of the network as well as to devise strategies to control their dynamics. Future work should investigate to what extent the predictions from the structural controllability can be extended to the dynamics of the networks undergoing structural changes.

## Additional Information

**How to cite this article**: Mengiste, S. A. *et al.* Effect of edge pruning on structural controllability and observability of complex networks. *Sci. Rep.* **5**, 18145; doi: 10.1038/srep18145 (2015).

## Supplementary Material

Supplementary Information

## Figures and Tables

**Table 1:** Some statistical quantities of the main networks used.

a. Conditioned pruning											
	Fig.	Network	*nE*	*nV*	*n*(*d* < 3)	*nCr*	*nCN*_0_	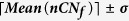			
ord.								out.	rnd.	rev.	
W-S	2.a.	**Small-world**, *p* = 2%	3,000	250	0	0	1	78 ± 3.5	207 ± 3.1	100 ± 3.6	22 ± 3.1
	2.b.	**Watts-Strogatz**, *p* = 10%	3,000	250	0	0	1	123 ± 3.1	198 ± 2.6	102 ± 4.3	21 ± 3.5
	2.c.	**Erdös-Rényi**, *p* = 100%	3,000	250	0	0	1	155 ± 4.4	192 ± 3.3	105 ± 4.8	5 ± 1.4
SF	2.d.	**Local attachement**	3,250	250	0	35	24	188 ± 3.0	180 ± 3.5	122 ± 3.9	24 ± 0.3
	2.e.	**Barabási-Albert**	3,081	250	0	28	39	205 ± 3.2	172 ± 2.9	136 ± 4.2	39 ± 0.0
	2.f.	**Duplication divergence**	3,151	250	1	0	146	210 ± 2.6	216 ± 4.1	166 ± 2.9	146 ± 0.0
BNN	2.j.	**Macaque**^21^	6,602	360	26	25	39	285 ± 3.3	273 ± 6.5	191 ± 4.5	51 ± 2.9
	2.k.	**Mouse**^22^	4,208	213	0	6	4	175 ± 3.3	172 ± 4.6	102 ± 3.5	20 ± 2.7
	2.l.	**C elegans**^23^	2,345	297	25	24	49	243 ± 3.2	208 ± 5.0	160 ± 4.0	58 ± 2.2
WS and SF (*N*, *ρ*)	S4.a.	**Random**, *n* = 250 **(2.c)**									
	S4.b.	**Random**, *n* = 500	12,584	500	0	0	1	335 ± 5.2	429 ± 3.6	208 ± 4.9	6 ± 2.1
	S4.c.	**Random**, *n* = 1000	50,082	1,000	0	0	1	699 ± 5.6	913 ± 4.1	416 ± 8.5	7 ± 2.3
	S4.d.	**Random**, *ρ* = 2%	1,336	250	3	7	3	147 ± 3.5	156 ± 4.1	106 ± 4.3	7 ± 1.9
	S4.e.	**Random**, *ρ* = 5% **(2.c)**									
	S4.f.	**Random**, *ρ* = 10%	6,301	250	0	0	1	163 ± 3.5	215 ± 2.3	104 ± 3.4	5 ± 1.6
	S4.g.	**Scale-free**, *ρ* = 1%	750	250	0	34	74	157 ± 2.9	129 ± 3.1	121 ± 4.4	74 ± 0.1
	S4.h.	**Scale-free**, *ρ* = 2%	1,250	250	0	35	48	171 ± 2.7	147 ± 3.5	121 ± 4.2	48 ± 0.1
	S4.i.	**Scale-free**, *ρ* = 5% **(2.d)**									
**b. Unconditioned pruning**											
											
Social	4, 2.g.	**Google + **24	1,506,896	211,187	121,867	51,680	78.50	42.52	31.51	5.29	
	4	**Amazon**^27^	1,234,877	262,111	12,469	8,458	48.78	49.06	27.94	10.27	
	4	**EU-email**^29^	420,045	265,214	248,706	245,791	92.78	43.08	40.82	2.32	
	4	**Peer-to-peer**^28^,^29^	147,892	62,586	38,520	46,227	23.09	49.70	13.63	5.53	
	4	**Wikipedia vote**^30^	103,689	7,115	2,960	4,736	60.76	23.11	9.03	1.16	
	4, 2.h.	**Facebook**^25^	88,234	4,039	256	568	62.67	47.25	16.35	1.97	
	4, 2.i.	**Airport**^26^	30,501	2,939	1,323	872	73.25	42.49	26.81	3.39	

The table summarises some graph-theoretical properties of the main networks that were investigated in conditioned (a) and unconditioned (b) pruning cases. *p* is the randomising probability of the connectivity of a ring lattice to get the desired Watts-Strogatz network. *ρ* and *n* are network density and size. *nE* and *nV* denote the number of edges and nodes in the network. *n*(*d* < 3) is the number of nodes in the network with both of their out- and in-degrees less than 3. *nCN*_0_ and *nCN*_*f*_ represent the number of controls before and after exhaustive pruning until a spanning directed tree (STD). *σ* represents the corresponding standard deviation for the 50 realizations. *ePDI* is an exhaustive pruning damage index. ⌈*x*⌉ is the smallest integer that is greater than or equal to *x*. The columns: ord., out., rnd. and rev. represent ordered-MM, out, random and resilient pruning strategies as in the Figure Legends (e.g. Figs 2 and 4). Because any unconditioned exhaustive pruning strategies finally result in no edge in the network, *nCN*_*f*_ = *nV*.
