# Supplementary material for: Effect of edge pruning on structural controllability and observability of complex networks
Source: Sci Rep. 2015 Dec 17;5:18145. doi: 10.1038/srep18145 (PMC4682190; doi:10.1038/srep18145)
Supplement: Supplementary Information [file srep18145-s1.pdf]

Supplementary information: Effect of edge pruning on structural controllability and observability of complex networks

Simachew Abebe Mengiste[1,2,*], Ad Aertsen[1], and Arvind Kumar[1,2,*]

[1]Bernstein Center Freiburg and Faculty of Biology, University of Freiburg, 79104 Freiburg, Germany.
[2]Computational Biology, School of Computer Science and Communication, KTH Royal Institute of Technology, Stockholm, 10044, Sweden
[*]AK: arvkumar@kth.se, SM: absimath@gmail.com

Extraction of different sets of controls

It is very useful to have different options of controls or sensors. For example, one or more nodes belonging to a set of control or sensor nodes may not be accessible for stimulation or recording. Without losing the minimality of control count, the existence of other choices makes it easier to consider a better set.

For a large network size, the number of different maximum matching sets is very large. Moreover, several maximum matching sets could result in the same set of control nodes. Therefore, one could use sampling via re-indexing [†] to extract distinct sets of control nodes for comparison. Moreover, such re-indexing could also be used to cope with a possible combinatorial explosion and estimate the likelihood of nodes to be included in a randomly chosen set of control nodes.

Enumeration of all maximum matchings of a bipartite graph is known.[1] Because a digraph can be treated as a bipartite graph (see the main text: **Maximum matching extraction algorithm**), the method is easily adaptable to our interest of control nodes identification. The algorithm takes the bipartite version G_b of the given digraph G with a maximum matching set, M_0. We also ignored the direction of the bipartite graph to avoid confusion as it is evident that edges are projecting from the source to the target pool. A maximum matching M_0 could be identified by the maximum flow algorithm described in the main text. The algorithm is then summarised as follows:

[†]re-indexing: $A \sim PAP^{-1}$ where P is the permutation matrix

Algorithm

Input: G_b, M_0

1. Color G_b such that all the edges in M_0 are red while all the remaining are black.

2. Check for an alternating cycle. If not, go to 5.

3. Choose any red edge e in the cycle C. Generate the new maximum matching set M_1 by replacing the red by the black edges of the cycle in M_0.
   For example, $M_0 = \{e_1, e_2, e_3, e_4\}$ and $C = \{c_1, e_2, c_2, e_3, c_1\} \Rightarrow M_1 = \{e_1, c_1, c_2, e_4\}$

4. Breakdown the problem in to two subproblems

   (a) Let $\bar{G}_b$ be the graph obtained from G_b by removing the nodes of e and all of their edges if any. With input $\bar{G}_b$, $M_0 - \{e\}$, go to 1.

   (b) Let $\hat{G}_b$ be the graph obtained from G_b by removing only the edge e.
   With input $\hat{G}_b$, M_1, go to 1.

5. Check for an alternating path of length 2 such that either the initial or the terminal node has no red edge. If no, end.

6. Choose the red edge e in the path. Generate the new maximum matching set M_1' by replacing the red by the black edge of the path in M_0.

7. Breakdown the problem in to two subproblems

   (a) Let $\bar{G}_b$ be the graph obtained from G_b by removing the nodes of e and all of their edges if any. With input $\bar{G}_b$, $M_0 - \{e\}$, go to 1.

   (b) Let $\hat{G}_b$ be the graph obtained from G_b by removing only the edge e.
   With input $\hat{G}_b$, M_1', go to 1.

In steps 4 and 7 of the above algorithm, the problem of maximum matching extraction is split into two simpler problems after a specific edge e is chosen: those that do and those that do not contain e in the following recursions. An illustration of this algorithm is presented in Supplemental Figure S1.

As an example, suppose there is a digraph G with all its edges E, given as $E = \{(n_1, n_2), (n_2, n_3), (n_3, n_4), (n_4, n_5), (n_2, n_5), (n_5, n_2), (n_4, n_3), (n_4, n_6)\}$. Then one can find a maximum matching set using the maximum flow approach, explained in the main text. One such set could be the subset with the first four elements, i.e. $M_0 = \{(n_1, n_2), (n_2, n_3), (n_3, n_4), (n_4, n_5)\}$. The above algorithm requires the graph G along with M_0 (see first panel of Supplemental Figure S1 (**b**)).

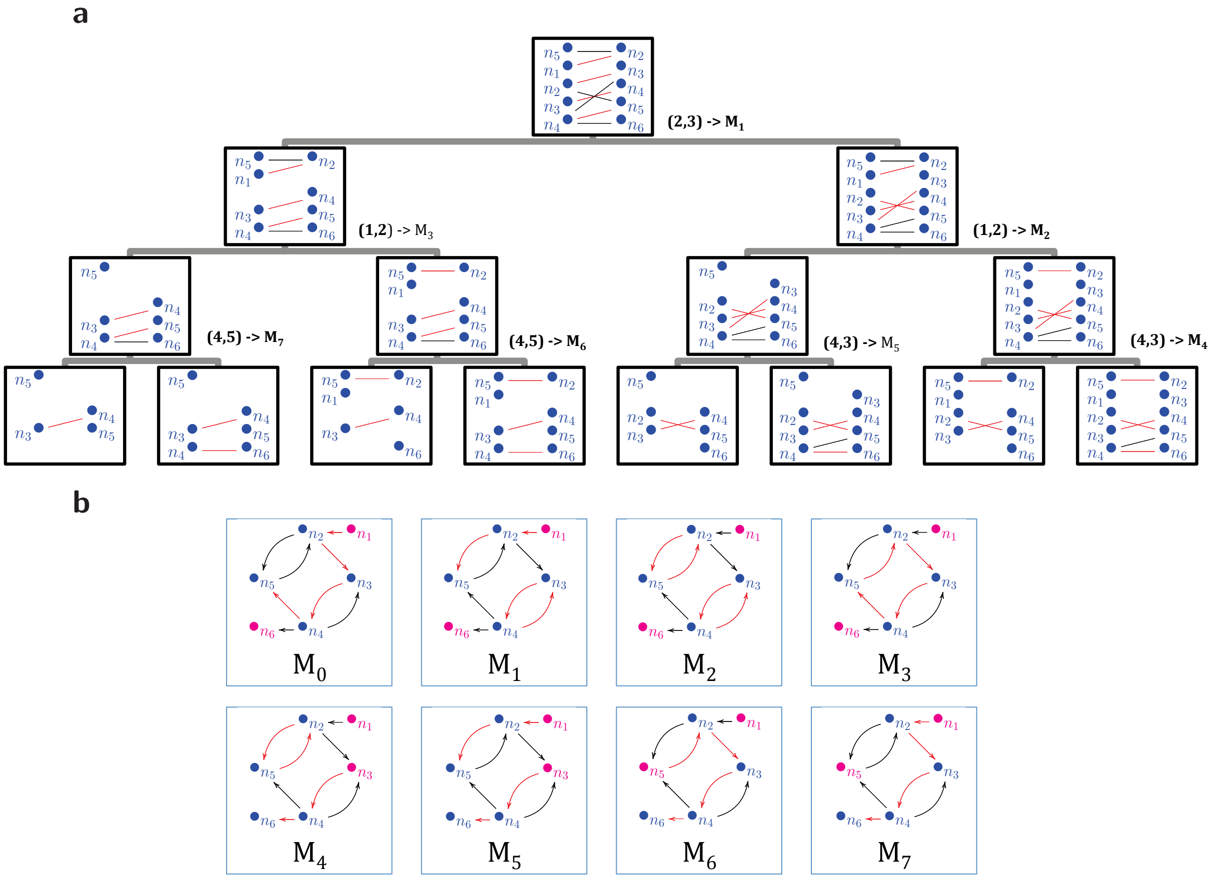

Supplementary Figure S1. Extraction of all possible sets of control nodes from the maximum matching set The parent of the tree in **a.** The example considers a digraph edge set $E = \{(n_1,n_2),(n_2,n_3),(n_3,n_4),(n_4,n_5),(n_2,n_5),(n_5,n_2),(n_4,n_3),(n_4,n_6)\}$. For this network, $M_0 = \{(n_1,n_2),(n_2,n_3),(n_3,n_4),(n_4,n_5)\}$ can be an MM set. **a**: Given the edge set E and a master MM set M_0, new MM sets, $M_1 \dots M_7$, are extracted at each branch of the tree using the bipartite version of the digraph. A branch is possible if there exists an alternating directed cycle (e.g. the parent bipartite graph of the tree contains such a cycle $\{n_5,n_4,n_3,n_2,n_5\}$) or a special path of length two (e.g. the child bipartite graph contains such a path (n_1,n_2,n_5)) (see the algorithm in section). Edges in red form a maximum matching set at each consideration. **b.** The digraph, all the eight maximum matchings (in red), and the corresponding control nodes (in magenta). The possible control nodes are $\{n_1,n_6\}$, $\{n_1,n_3\}$ and $\{n_1,n_5\}$.

Network generation procedures

All networks studied here are without self connections and assumed to have non-overlapping nodes. As described in the main text, both experimental and artificial network data have been investigated for the susceptibility of their control profile to edge pruning. The main synthetic complex random networks included scale-free networks and networks with high, intermediate and low small-world indices. In this section, we describe how these synthetic networks were generated.

Small-world, random and intermediate networks

We adapted the Watts-Strogatz mechanism of generating different graphs to digraphs.

- Step 1: **Generate a ring lattice**

  According to the desired density, we generated a ring network of N nodes with k degree each. The edges were oriented in

one direction so that the network had a circulant adjacency matrix.

- Step 2: **Randomise targets**

  With a chosen probability p, we randomised the targets of each edge.

- Step 3: **Randomise sources**

  Similarly, the sources of edges were rewired with probability p.

Small-World networks are characterised by low average path-length and high clustering coefficients. For an undirected network, there is always a finite average path-length. However, it is common to find a node in a directed network that cannot reach some nodes in any number of steps. It is difficult to characterise a network with small-world features without a finite distance between each pair of nodes. The initial clockwise or counter-clockwise flow of edges in the first step gets rid of this problem as it is clearly a strongly connected network. A network with high small-world index could be formed by randomising the edges of the ring lattice with a small probability. A small probability to randomize the connections preserves the high clustering, unless it is as sparse as a simple ring network. We chose $p = 0.02, p = 0.1$ and $p = 1$ to create small-world (SW), intermediate (W-S) and random (E-R) networks, respectively.

As the second step randomises the targets of the edges, the out-degree of the nodes remains unaffected. Because randomisation at the end of this step is half-complete, the rewiring of the sources was performed in a similar manner (step 3).

Scale-free networks

Scale-free network generation models share similar procedures: the network expands in size following a rule from a small network such that the degree distribution of the resulting network follows a power law. The three models to generate scale-free networks were adopted using the procedure as in Ruths and Ruths.[2]

- **Barabasi-Albert**

  In this algorithm, the network is created according to a growth process by systematically adding new nodes. Based on the final size N and the total number of edges nE of the desired network, the number of edges m a new node adds to the network is equal to the average degree of the network. The generation begins by few m connected nodes. For the remaining, progressive addition of $N - m$ nodes, the m neighbours of the new node are determined by the degree distribution of the existing nodes in rich-gets-richer basis. That is, the higher the degree of a node, the more likely it is to become connected to the newly added nodes.

- **Local Attachement**

  Similar to the Barabasi-Albert model, we first fixed the size of the network N and the number of edges m that a new node adds to the existing network. The procedure begins with a full network of size m without self-connections. To extend the

size to N, we added $N-m$ nodes one by one. For each newly added node, r nodes were randomly selected from the existing nodes (the parent nodes – P). Next, $m-r$ nodes (O) were selected at random from all out-neighbours of P that were not in P. The new node was then connected to the m nodes belonging to the set $P \cup O$. This addition was repeated in the same procedure until the size reached N.

- **Duplication Divergence**
  By fixing the size of the network N and the probability p of edge duplication, we started the network with a pair of interconnected nodes without self-edges. Then, for each newly added node (n) of $N-2$ nodes, a random node k was chosen from the existing network and each out-neighbor and in-neighbor of k became an out-neighbor and in-neighbor of n, respectively, with probability p. In case no edge was duplicated for the new node, the node was deleted and the process was repeated. This process was iterated until the desired size N was attained.

Alternative implementations of resilient pruning

Different implementations of resilient pruning exist. Their effect is similar, although they differ in computational costs.

- **Random testing:** In this procedure a random edge is selected and removed if it does not cause network fragmentation and a change in the number of controls. The process is repeated until every edge has been checked. After that, pruning for the next larger number or random pruning without fragmentation could be performed to reach a minimum spanning directed tree (MSDT). In most cases, the edges that show a change from the original size in this procedure are only a few.

- **Reverse-ordered pruning:** This method was described in the main text and was used to generate the results throughout this manuscript. Whereas ordered-MM pruning tries to destroy the edges that have larger relative rank, this method starts with edges with the least tendency of belonging to a first generation maximum matching set. Therefore, the deletion of such edges does not affect the original number of controls for almost the entire pruning. This method is computationally cheap compared to the former. One could use a mid-point approach, since a change in the control count is mostly less likely at 50% of edge deletion in this procedure.

- **Fixed MM - based pruning:** This procedure starts with a maximum matching and supplements it with a few more edges to form an MSDT and protects the tree from deletion. This is not as flexible as the first two methods because it relies on a specific maximum matching set.

Pruning of networks with different size and density

Even though the network size and connection density affect the dynamical properties of the network,[8] the efficacy of different pruning strategies in changing the controllability profile is largely independent of the size of the random networks (Supplemental Figure S2 a-c). With increasing connection density, the order of efficacy of pruning strategies was also preserved (Supplemental Figure S2 d-f). Note that, at higher connection density the difference between the out- and ordered MM pruning increased,

because of the associated increase in its small-world index. In other words, the denser the random network, the higher the clustering coefficient and the lower the average path length. This implies that the maximum matching sets have smaller cardinality, forcing ordered-MM pruning to have a relatively smaller effect in increasing the control count.

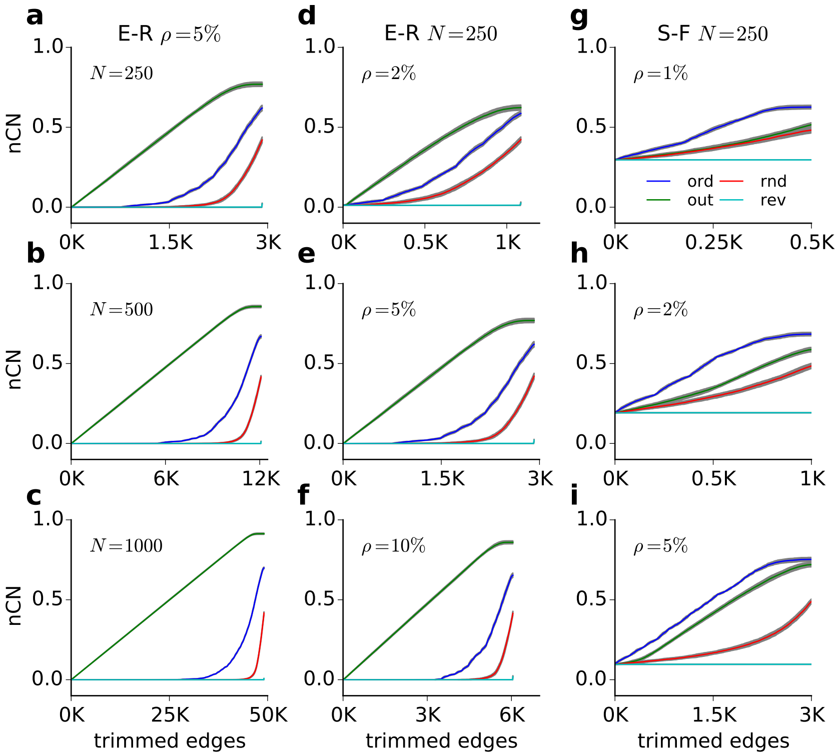

Supplementary Figure S2. The pruning principles on networks of different size and density
The color description is as in figure 2 of the main text. The first six subfigures (**a-f**) are Erdos-Renyii networks where the first three fixes the density to 5% while changing the size, 250, 500, 1000 (**a-c**), where as the next three fixes the size to 250 while changing the density, 2%, 5%, 10% (**d-f**). The subfigures in the last column, (**g-i**), are scale-free networks with fixed size (N=250) but different density (1%, 2%, 5%) generated by the local preferential attachment model. In increasing size (**a-c**) and density (**d-f**), random networks react in a similar order to the pruning strategies. As the density increases in scale-free networks (**g-i**),out- pruning deviates from random pruning more. As a network gets denser, ordered pruning performs less and gets closer to random pruning due to the more Small-World feature it gains by the additional edges.

Sorted out-pruning

Outpruning has been considered so far on the nodes of networks without any order (see Fig. 2, Fig. 3 in the main text and Supplemental Figure S2). In other words, a node was selected at random and its outgoing edges were deleted exhaustively one by one with or without fragmenting the network. The pruning was repeated for each node.

In this pruning strategy, nodes were sorted according to their degree, out-degree and in-degree before pruning. We refer to such out-pruning of edges from nodes in order of increasing as well as decreasing order of their degree as *sorted out-pruning*.

In the earlier unsorted out-pruning cases, the error regions (gray) bounding the mean of the out-pruning (green) of 50 different realisations were smaller (Supplemental Figure S3). However, in sorted out-pruning, six different sortings could result in large differences in the control profile of the network (Supplemental Figure S3). It is unlikely that a sorted out-pruning would be selected in a random selection. Even if they were selected, their effect would be highly masked by the remaining realisations that do not show differences among each other that much.

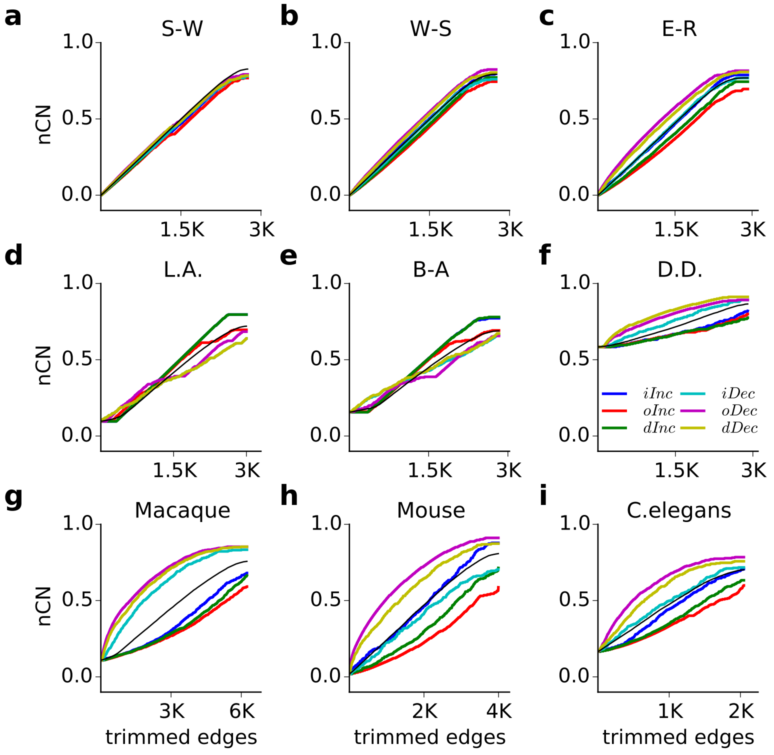

Supplementary Figure S3. **Outpruning with degree sorted nodes**
The networks are the same as the first nine networks treated in Fig. 2 of the main text. Unlike performing out-pruning by randomly picking a node at a time, here, nodes are sorted according to their in-degree, out-degree and degree in increasing as well as decreasing order for out-pruning. In the color description in **b**, *iI, iD, oI, oD, dI* and *dD*, the first letter '*i*', '*o*' or '*d*' stands for in-degree, out-degree or degree. The last letters *I*' and '*D*' represent increasing and decreasing orders. For example, '*iI*' means that out-pruning is performed on outgoing edges from nodes of least out-degree to nodes of most out-degree. The colors blue, cyan, red, pink, green and yellow correspond to *iI, iD, oI, oD, dI* and *dD*. The thin black trace corresponds to the mean out-pruning also shown in green in Fig. 2 of the main text. Although the error region for the unsorted out-pruning in Fig. 2 appears to be little, sorted out-pruning may show significant deviations from each other **g-i**.

Random pruning of biological neuronal networks

Here, we investigated the experimental neuronal connectivity data of five different organisms, namely long distance connectivity of the Macaque brain,[3] the neuronal connectivity of the nematode *C. elegans*,[4] the cortico-cortical connectivity of the cat brain,[5] the macroscopic connectivity of the pigeon brain,[6] and the mesoscale connectome of the mouse brain.[7]

Of all pruning strategies and networks that we considered in this study, random pruning turned out to cause an exponential growth of the control count with a decreasing average degree in the BNNs (see Supplemental Figure S4).

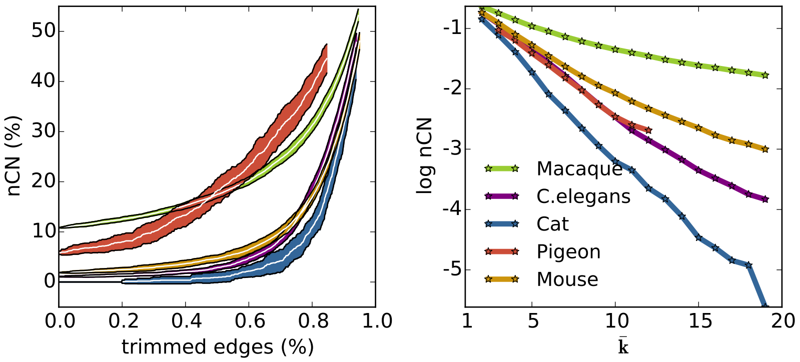

Supplementary Figure S4. The effect of random pruning on five biological neuronal networks. Left: Random pruning of the five biological neuronal networks (BNNs): macaque, *C. elegans*, cat, pigeon and mouse. The horizontal axis represents the fraction of deleted edges, whereas the vertical axis represents the percentage of the numbercontrol of controls. **Right:** The horizontal axis is the average degree of the network corresponding to the deletion in the left, but flipped. The vertical axis corresponds to the logarithmic values of the percentages in the left. All of the five investigated BNNs show that random pruning results in an exponential dependence of their number of controls on the average degree of the network.

Pruning without fragmenting the network

As described in the main text, this is a conditioned pruning where an edge deletion strategy is not allowed to fragment the network. This variant has been considered to keep a network intact throughout the pruning process so that we have a single network at any stage of pruning, in contrast to the unconditioned pruning.

Hub-based spanning tree

A hub-based spanning tree tries to generate an spanning tree with few hubs. If there are few rich hubs that could span the network through their projections, many leaf and source nodes are expected in the tree. This imposes the requirement of many controls or sensors.

Let $v_1,\dots,v_n$ be degree-sorted vertices of a given graph G, i.e. $degree(v_i) \geq degree(v_j)$ whenever $i < j$. If $G(v)$ denotes the subgraph of G containing the vertex v with all its neighbours and edges and $\cup$ denotes the union of subgraphs, the algorithm can be stated as follows:

Algorithm

Input: $v_1,\dots,v_n$, vertices of G, sorted with respect to their degree

1. Consider $H := G(v_1)$
2. Check if H is a spanning directed tree. If yes, go to 5.
3. Choose unvisited v_k such that $H \cup G(v_k)$ contains the greatest number of nodes.
4. With $H := G(v_1, v_k)$, go to 2.

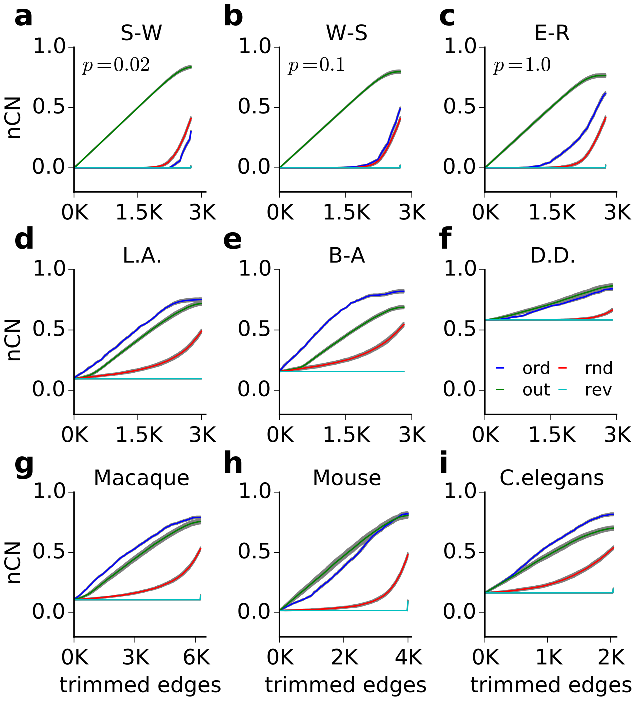

Supplementary Figure S5. Conditioned pruning This is similar to the nine subfigures of the second figure in the main text, (Fig 2. a-f, j-l). The only difference is that in exhaustive conditioned pruning, we did not fragment the network for each of the pruning strategies. That is, the pruning in this case was done up to a spanning directed tree. The comparison on the performance of the pruning strategies is similar to the unconditioned case (Fig. 2 a-f, j-l).

5. Randomly prun the SPD to an MSDT

This process resulted in a spanning tree that requires more controls than spanning tree preferences of any of the pruning methods considered. (see **Conditioned pruning** in the main text.)

The effect of the pruning strategies on the network structure

In this section, a node in a complex network is referred to as a *few-degree node* if both its out- and in-degrees are less than three. A subgraph in a digraph (directed network) is *strongly connected component(SCC)* if there is a directed path from any node to any other node in the subgraph.

The number of SCC ($nSCC$) and the number of few-degree nodes ($n(deg < 3)$) have been recently linked to or used as estimates for the number of controls (nCN).[9–11] Liu. et al 2013 commented that $nSCC$ estimates nCN well for non-linear systems but underestimates it in linear systems due to dilation. Menichetti et. al 2014 claimed that the density of nodes with in-degree and out-degree equal to one and two determines the number of driver nodes in the network.

To check the validity of the claims in some of the networks, we studied how the four pruning strategies shape the relationships between nCN and $nSCC$ as well as between nCN and $n(deg < 3)$. Our result, summarized in Fig. S6, showed that neither of the networks we considered for this demonstration resulted in expected relationship. Resilient pruning strategy strongly contradicts the estimation of nCN by either of the two quantities because it resulted in an alarming change in both $nSCC$ and $n(deg < 3)$

for the same number of controls. Moreover, a network with a specific *nSCC* could keep changing its *nC* (cf. Fig. S6c. In fact, if the claims had been valid to the networks we have analyzed, the relationship should have been preserved regardless of the type of the pruning strategy. We noted that although the claims may be valid for many networks, there are such exceptions as the networks analyzed here.

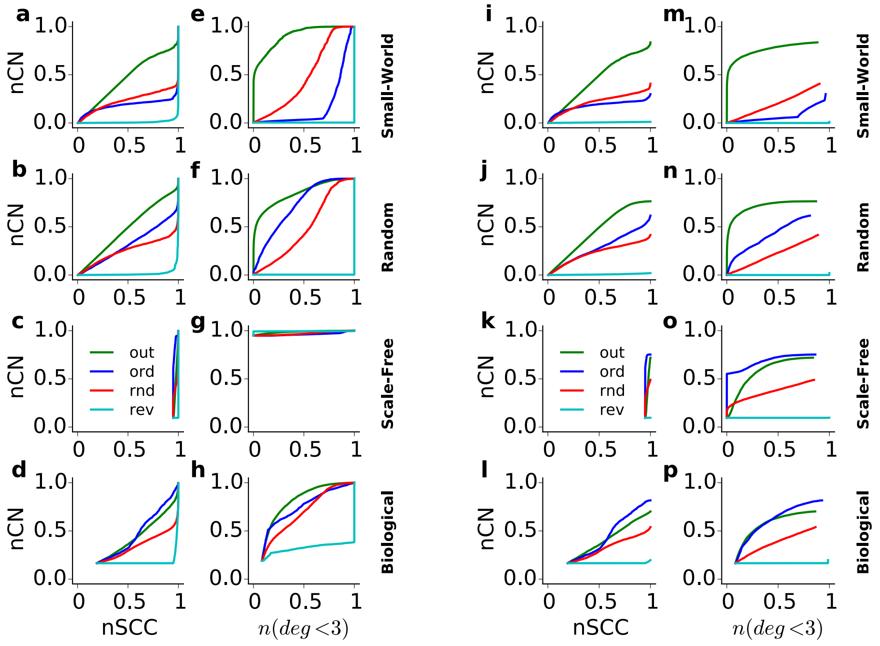

i. Unconditioned pruning *ii.* Conditioned pruning

Supplementary Figure S6. **The relationship between nSCC, n(d < 3) and nCN** The four networks in row are the same networks used in Fig. 2 **a,c,d** and **i** - corresponding to the small world, Erdos-Renyii, Local-Attachment and the c-elegans neural connectome. While the left half (*i.* **a-h**) is for unconditioned pruning, the right half (*ii.* **i-p**) is for conditioned pruning. Different pruning strategies relate the increase in control size *nCN* with the increase in the number of strongly connected components *nSCC* (**a-d, i-l**) and with the increase in the number of few degree nodes $n(d < 3)$ (**e-h, m-p**) differently. It shows that *nSCC* or $n(d < 3)$ can not predict the *nCN* alone.

References

1. Uno, T. *Algorithms for enumerating all perfect, maximum and maximal matchings in bipartite graphs* (Springer, 1997).

2. Ruths, J. & Ruths, D. Control profiles of complex networks. *Science* **343**, 1373–1376 (2014).

3. Modha, D. S. & Singh, R. Network architecture of the long-distance pathways in the macaque brain. *Proc Nat Acad Sci* **107**, 13485–13490 (2010).

4. Watts, D. J. & Strogatz, S. H. Collective dynamics of small-world networks. *Nature* **393**, 440–442 (1998).

5. Hilgetag, C.-C., Burns, G. A., O'Neill, M. A., Scannell, J. W. & Young, M. P. Anatomical connectivity defines the organization of clusters of cortical areas in the macaque and the cat. *Philos. Trans. R. Soc. Lond., B, Biol. Sci.* **355**, 91–110 (2000).

6. Shanahan, M., Bingman, V. P., Shimizu, T., Wild, M. & Güntürkün, O. Large-scale network organization in the avian forebrain: a connectivity matrix and theoretical analysis. *Front. Comput. Neurosci.* **7** (2013).

7. Oh, S. W. *et al.* A mesoscale connectome of the mouse brain. *Nature* **508**, 207–214 (2014).

8. Kumar, A., Schrader, S., Aertsen, A. & Rotter, S. The high-conductance state of cortical networks. *Neural Comput* **20**, 1–43 (2008).

9. Liu, Y.-Y., Slotine, J.-J. & Barabási, A.-L. Observability of complex systems. *Proc Nat Acad Sci* **110**, 2460–2465 (2013).

10. Nie, S., Wang, X., Zhang, H., Li, Q. & Wang, B. Robustness of controllability for networks based on edge-attack. *PLoS One* **9**, e89066 (2014).

11. Menichetti, G., DallÁsta, L. & Bianconi, G. Network controllability is determined by the density of low in-degree and out-degree nodes. *Phys. Rev. Lett* **113**, 078701 (2014).
